# The use of ketamine in the management of suicidal crisis: a literature review

**DOI:** 10.1192/j.eurpsy.2026.11402

**Published:** 2026-07-31

**Authors:** A. Rami

**Affiliations:** Razi Hospital, Manouba, Tunisia

## Abstract

**Introduction:**

Suicidal crisis represents a major psychiatric emergency, often occurring in the context of mood disorders and requiring rapid, effective interventions. Traditional antidepressant treatments usually have a delayed onset of action, underscoring the need for fast-acting therapeutic alternatives. In recent years, ketamine—an NMDA receptor antagonist—has emerged as a promising option for the rapid reduction of suicidal ideation.

**Objectives:**

This literature review aims to synthesize current evidence on the efficacy, mechanisms, and safety of ketamine and its enantiomer esketamine in the management of acute suicidal crises.

**Methods:**

A systematic search of recent peer-reviewed studies, meta-analyses, and clinical guidelines (2015–2025) was conducted using PubMed, Scopus, and ScienceDirect. Articles assessing both intravenous ketamine and intranasal esketamine in suicidal patients were included.

**Results:**

The reviewed literature consistently demonstrates that a single subanesthetic dose of intravenous ketamine (0.5 mg/kg) produces a **rapid reduction in suicidal ideation within hours**, with effects typically lasting up to one week. Esketamine nasal spray, approved for treatment-resistant depression, also shows significant short-term anti-suicidal effects when used alongside standard care. Neurobiological findings suggest that ketamine’s benefits involve **glutamatergic modulation**, **enhanced synaptic plasticity**, and downstream effects on **BDNF signaling** and **AMPA receptor activation**. However, heterogeneity in dosing protocols, short duration of benefit, and potential dissociative or cardiovascular side effects remain important limitations.

**Conclusions:**

Ketamine represents a major paradigm shift in the acute management of suicidal crises, offering **rapid-onset relief** where conventional antidepressants fail. Nevertheless, its **transient efficacy** and the need for **specialized monitoring** highlight the importance of integrating ketamine into **multimodal, safety-focused treatment strategies**. Future research should clarify optimal dosing schedules, maintenance strategies, and long-term outcomes to fully establish ketamine’s role in suicide prevention.

**Disclosure of Interest:**

None Declared

